# Does the Degree of Hepatocellular Carcinoma Tumor Necrosis following Transarterial Chemoembolization Impact Patient Survival?

**DOI:** 10.1155/2016/4692139

**Published:** 2016-02-02

**Authors:** Nathan Haywood, Kyle Gennaro, John Obert, Paul F. Sauer, David T. Redden, Jessica Zarzour, J. Kevin Smith, David Bolus, Souheil Saddekni, Ahmed Kamel Abdel Aal, Stephen Gray, Jared White, Devin E. Eckhoff, Derek A. DuBay

**Affiliations:** ^1^University of Alabama at Birmingham School of Medicine, Birmingham, AL 35233, USA; ^2^Biostatistics Division, School of Public Health, University of Alabama at Birmingham, Birmingham, AL 35233, USA; ^3^Department of Radiology, University of Alabama at Birmingham, Birmingham, AL 35233, USA; ^4^Liver Transplant and Hepatobiliary Surgery, University of Alabama at Birmingham, Birmingham, AL 35233, USA

## Abstract

*Purpose*. The association between transarterial chemoembolization- (TACE-) induced HCC tumor necrosis measured by the modified Response Evaluation Criteria In Solid Tumors (mRECIST) and patient survival is poorly defined. We hypothesize that survival will be superior in HCC patients with increased TACE-induced tumor necrosis. * Materials and Methods*. TACE interventions were retrospectively reviewed. Tumor response was quantified via dichotomized (responders and nonresponders) and the four defined mRECIST categories.* Results*. Median survival following TACE was significantly greater in responders compared to nonresponders (20.8 months versus 14.9 months, *p* = 0.011). Survival outcomes also significantly varied among the four mRECIST categories (*p* = 0.0003): complete, 21.4 months; partial, 20.8; stable, 16.8; and progressive, 7.73. Only progressive disease demonstrated significantly worse survival when compared to complete response. Multivariable analysis showed that progressive disease, increasing total tumor diameter, and non-Child-Pugh class A were independent predictors of post-TACE mortality.* Conclusions*. Both dichotomized (responders and nonresponders) and the four defined mRECIST responses to TACE in patients with HCC were predictive of survival. The main driver of the survival analysis was poor survival in the progressive disease group. Surprisingly, there was small nonsignificant survival benefit between complete, partial, and stable disease groups. These findings may inform HCC treatment decisions following first TACE.

## 1. Introduction

Transarterial chemoembolization (TACE) is indicated for patients with hepatocellular carcinoma (HCC) who are not candidates for transplantation, resection, or ablation [1, 2]. TACE is the most common oncologic treatment for HCC patients with Medicare in the United States [3]. The Scientific Registry of Transplant Recipients (SRTR) shows that TACE is the most common bridging therapy for waitlisted liver transplant patients with HCC [3, 4]. The American Association for the Study of Liver Disease (AASLD) current HCC treatment recommendations state that “TACE is recommended as first line non-curative therapy for non-surgical patients with large/multifocal HCC who do not have vascular invasion or extrahepatic spread” [5, 6].

The goal of TACE is to induce HCC tumor necrosis via occlusion of tumor arterial blood flow along with local administration of cytotoxic chemotherapy [1]. Tumor necrosis is estimated via changes in HCC tumor arterial enhancement on post-TACE imaging, as quantified by the modified Response Evaluation Criteria In Solid Tumors (mRECIST) [7–9]. There are four categories of tumor response according to mRECIST: complete response, partial response, stable disease, or progressive disease [6, 8–10]. Studies have examined the correlation between post-TACE radiologic assessment and actual tumor necrosis on explant pathology [11–16]. However, relatively few studies have examined the correlation between radiologic evaluation of tumor necrosis and post-TACE survival [7, 8, 17].

A study by Memon et al. showed a correlation between radiologic evaluation of HCC tumor necrosis following locoregional therapies (including TACE and Y90) and overall survival in Child-Pugh classes A and B7 patients [17]. This study estimated HCC tumor necrosis via the European Association for the Study of Liver (EASL) response criteria [17]. A study by Kim et al. demonstrated that tumor response grading by mRECIST was predictive of overall survival in Child-Pugh classes A and B patients who underwent TACE for HCC [7]. Studies by Prajapati et al. and Gillmore et al. have also demonstrated that mRECIST response criteria are predictive of overall survival in HCC patients following TACE [18, 19]. However, in each of these analyses, patients were dichotomized into responders (complete and partial response mRECIST categories) or nonresponders (stable and progressive disease mRECIST categories) [7, 18, 19]. Few studies have shown that the 4 mRECIST categories are predictive of overall survival and in each case, the patient population was limited to only Child-Pugh class A or B patients [20–22]. Accordingly, there are two identified knowledge gaps in the literature regarding the impact of TACE-induced HCC necrosis and survival: the first is assessing patients with compromised liver disease (Child-Pugh class B8 or worse) and the second is to measure the difference between survival outcomes between each of the four mRECIST categories.

The purpose of this study was to measure the association between TACE-induced HCC tumor necrosis and survival in HCC patients with Child-Pugh class A, B, or C, measured by dichotomized (responder and nonresponder) and distinct (complete, partial, stable, and progressive) mRECIST categories. It is hypothesized that survival will be superior in HCC patients with increased TACE-induced tumor necrosis.

## 2. Methods

The research protocol for this study was approved by the UAB institutional review board. A retrospective chart review was performed for all patients receiving a TACE at UAB between January 2008 and April 2014. Methods presented here were adapted from previous studies [23].

### 2.1. Patient Population

Patients were diagnosed with HCC according to the AASLD criteria. The decision to offer TACE to patients with HCC was made by a multidisciplinary liver tumor board at UAB including medical oncologists, surgeons, hepatologists, and interventional radiologists. Patient candidacy for TACE was guided by established AASLD practice guidelines [5, 6]. One exception was the inclusion of highly selected Child-Pugh C candidates with single enhancing peripheral HCC tumors felt to be “easy” TACE procedures by the interventional radiologists.

A list of consecutive patients treated with a first TACE was generated from the UAB Interventional Radiology procedures electronic database. TACE patients were excluded if they had non-HCC tumor type. Recurrent HCC following liver resection or transplantation also were excluded. In addition, HCC tumors that had previously been treated with another locoregional therapy such as radiofrequency ablation or external beam radiotherapy were excluded. Patients who received liver transplantation following TACE were censored at the time of transplant. Those who received multiple TACE procedures prior to assessing tumor response were excluded.

### 2.2. HCC Tumor Assessment and Post-TACE Tumor Necrosis Quantification

#### 2.2.1. HCC Diagnosis

HCC is diagnosed according to AASLD criteria: when there is an arterially enhancing lesion with portal venous washout and/or pseudocapsule formation on delayed phase seen on multiphase contrast enhanced Computed Tomography (CT) or dynamic contrast enhanced Magnetic Resonance Imaging (MRI) of the liver [24, 25].

#### 2.2.2. HCC Tumor Necrosis (Figure 1)

Tumor response was assessed via the modified Response Evaluation Criteria in Solid Tumors (mRECIST). In 2008, the AASLD modified the National Cancer Institute RECIST criteria to unify assessment of radiographic response for hepatocellular carcinoma [8]. The modified RECIST criteria for tumor response are based on measurement of reduction in viable enhancing tumor in the arterial phase of dynamic CT or MRI imaging, rather than purely tumor shrinkage measured by the greatest diameter of the lesion. There are four categories of tumor response according to mRECIST: complete response, partial response, stable disease, or progressive disease [8]. Complete response is defined as the disappearance of tumor arterial enhancement. Partial response is defined as at least 30% decrease in the longest diameter of arterial enhancement. Stable disease is defined as a response that did not fall into the partial response or progressive disease category. Progressive disease is defined as growth of at least 20% of the sum of the longest diameter of the lesions. Electronic calipers were used to measure the longest diameter of arterial enhancement of the index lesion in the axial plane. Both MRI and CT imaging modalities were used to quantify HCC tumor necrosis. The tumor response used for statistical analysis was measured one month following initial TACE procedure for all patients included in this study.

### 2.3. TACE Protocol

The decision to offer TACE as locoregional oncologic therapy for patients with HCC was made at the UAB multidisciplinary liver tumor board. Over the course of this study, Lipiodol-based TACE was the most common approach initially, whereas most TACE procedures currently are performed with drug eluting beads (DEBS). Lipiodol-based TACE consisted of HCC embolization with a mixture of Lipiodol, 50 mg Doxorubicin, and 400 *μ*m Embozene® microspheres (Celonova, USA). DEBS TACE consisted of either LC beads (Biocompatibles, UK) or QuadraSpheres® expanding microspheres (BioSphere Medical, France). These beads were eluted with 50 mg Doxorubicin. As a general strategy, selective targeted embolization was routinely done for focal lesions. In cases of multifocal disease, lesions larger than >2-3 cm were selectively targeted followed by lobar embolization if necessary.

### 2.4. Data Analysis

Patient demographics, clinical history, laboratory data, and cross sectional imaging characteristics were collected. Pre-TACE imaging CT and MRI variables include number of lesions, size of tumors, and sum of axial diameters of the 3 largest tumors in the case of multifocal HCC. Data collected from post-TACE CT and MRI imaging included HCC tumor necrosis measured according to mRECIST criteria [8]. To allow common statistical procedures, the analysis was restricted to examination of the index HCC tumor that was defined as the largest tumor (if more than one tumor per patient had been used in the analysis, the common assumption of independent data observations would have been violated). Analysis of Variance was used to compare means among mRECIST groups. The primary analytic approach for testing association between mRECIST and categorical variables utilized Chi-square analyses. Kaplan-Meier curves were constructed to evaluate patient survival. Survival probabilities were analyzed with the Wilcoxon test since it is more sensitive to detect differences at shorter survival times. Cox Proportional Hazard Regression was used for a multivariable adjusted analysis and to estimate survival curves adjusting for demographic and clinical baseline characteristics. For all inferences, the probability of a Type I error (*α*) was set to 0.05. All analyses were conducted using the SAS 9.4 (Cary, NC).

## 3. Results

### 3.1. Patient Demographics

Between January 2008 and April 2014, 317 consecutive patients received a first TACE at UAB and were included in this study. The study population included Child-Pugh class A (39%), Child-Pugh class B (51%), and Child-Pugh class C (10%) patients. The most common etiologies of liver disease were hepatitis C virus (49%) alcohol (25%) and nonalcoholic steatohepatitis (19%). Tumor response was evaluated via MRI for 75 patients and CT for 242 patients. In total, 33 patients were treated with Sorafenib prior to TACE and 53 patients were treated with Sorafenib following TACE.

### 3.2. HCC Tumor Necrosis

**Figure 2:**
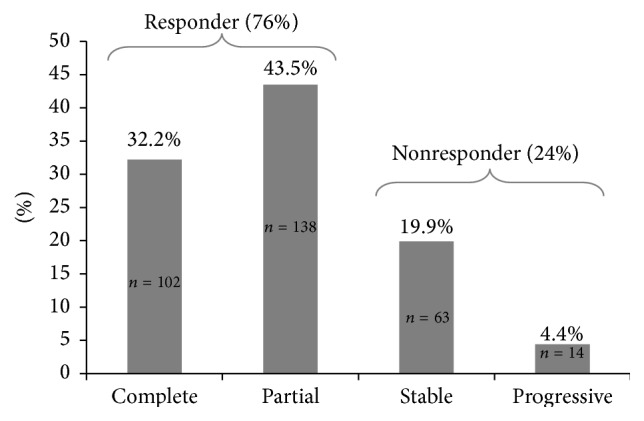
Distribution of HCC tumor necrosis following TACE as quantified by the mRECIST criteria.

HCC tumor necrosis distribution is shown in Figure 2. Patients were more likely to be responders (76%) than nonresponders (24%). Individual mRECIST category analysis revealed that the most common response to TACE was partial (43.5%), followed by complete (32.2%) and stable (19.9%). The least frequent mRECIST response was progressive disease (4.4%).

Population basic demographics, stratified by mRECIST category, are presented in Table 1. There were no significant differences in the distribution of age, gender, race, or Child-Pugh class and mRECIST response. The prevalence of hepatitis C virus varies among the different mRECIST groups, with a higher prevalence in the complete and partial response groups. There was a significant association between worse mRECIST response and both increasing HCC tumor number (*p* = 0.003) and increasing max tumor diameter (*p* < 0.0001). A similar association was observed between total axial diameter of the 3 largest HCC tumors and worse mRECIST response (*p* < 0.0001). The mRECIST response was predictive of repeat TACE (*p* = 0.025). Patients with stable disease were the most likely to undergo repeat TACE (55.2%) while patients in the complete response were the least likely to undergo TACE (35.6%).

### 3.3. Survival Outcomes

**Figure 3:**
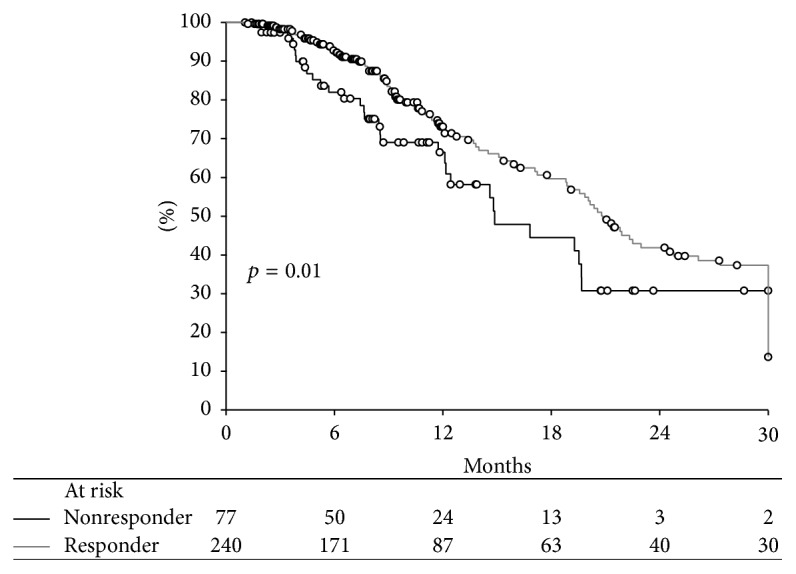
Unadjusted post-TACE survival as a function of mRECIST dichotomized into responders (complete and partial response) and nonresponders (stable and progressive disease).

**Figure 4:**
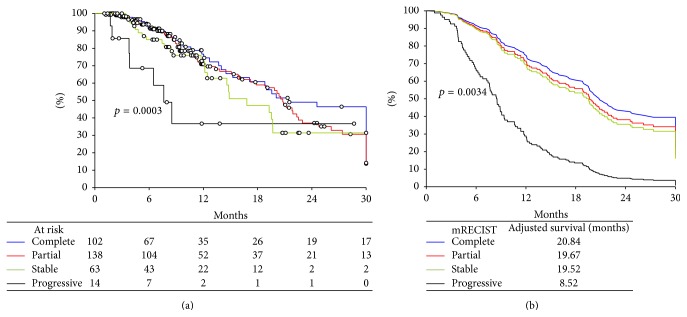
(a) Crude, unadjusted post-TACE survival as a function of mRECIST category. (b) Adjusted post-TACE survival as a function of mRECIST category.

Univariate and subsequent multivariable analyses were carried out to examine post-TACE survival as a function of mRECIST response. Kaplan-Meier curves were constructed for both the dichotomized, responders and nonresponders (Figure 3), and the 4 individual mRECIST groups (Figure 4(a)).

The survival analysis investigating mRECIST categories dichotomized into responders (complete and partial response) and nonresponders (stable and progressive disease) shows patient survival significantly varied according to the dichotomized mRECIST response (Figure 3). Median survival was significantly longer in responders than in nonresponders (20.8 months versus 14.9 months, *p* = 0.011).

Additional analyses were then carried out to examine survival in the four defined mRECIST categories. Crude, unadjusted survival analysis shows that patient survival following TACE varied according to mRECIST category (*p* = 0.0003, Figure 4(a)). Patients with a complete response had the longest median survival (21.44 months), followed by partial response (20.78 months) and stable response (16.82 months), while patients with progressive disease showed the shortest median survival (7.73 months).

A multivariate analysis of patient and HCC tumor predictors of post-TACE survival was performed (Table 2). There was no significant association between post-TACE survival and age, gender, or race. Child-Pugh class was significantly associated with post-TACE survival (*p* < 0.001). Compared to Child-Pugh A patients, Child-Pugh B [HR 2.67, 95% CI (1.8, 3.94), *p* < 0.001] and Child-Pugh C [HR 2.26, 95% CI (1.09, 4.65), *p* = 0.028] have significantly increased mortality risk. Increased total tumor diameter was also significantly associated with decreased post-TACE survival [HR 1.04/cm of increasing tumor diameter, 95% CI (1.01, 1.08), *p* = 0.009]. The mRECIST response was also significantly associated with post-TACE survival (*p* = 0.003). However, when using complete response as a reference, there was no significant survival difference in partial and stable disease categories. Only the progressive disease category was significantly associated with decreased post-TACE survival (HR 4.99, 95% CI (1.19, 11.09), *p* < 0.001).

Adjusted survival curves were constructed adjusting for statistically significant covariates. Again, post-TACE survival was significantly associated with the four defined mRECIST categories (*p* = 0.003, Figure 4(b)). Adjusted median survival estimates were greatest in patients with a complete response (20.84 months), followed by partial response (19.67 months) and stable response (19.52 months), while patients with progressive disease showed the shortest median survival (8.52 months).

## 4. Discussion

Similar to published reports [7, 17–22], this study demonstrates a significant survival advantage in responders compared to nonresponders following TACE for HCC. In isolation, this finding suggests that patients who exhibit a complete or partial response to TACE will experience approximately 6 months increased survival compared to patients with stable or progressive disease. To further investigate the association between TACE-induced HCC tumor necrosis and patient survival, we conducted survival analyses of the four defined mRECIST categories.

Patients with complete response experienced the longest median survival followed by patients with partial response and stable disease. Much to our surprise, there was only a small, nonstatistical difference in survival outcomes between patients with complete, partial, and stable disease mRECIST responses. Compared to complete response, the only statistically different survival outcome following TACE was observed in the progressive disease category. Patients with progressive disease experienced greatly decreased survival when compared to those in other mRECIST categories. The most important finding from this study is that the poor survival in the progressive disease group is the main statistical driver of the dichotomous (responder and nonresponder) and the four defined mRECIST group survival benefit analysis.

Findings presented here are congruent with previous works that have shown association between radiologic evaluation of tumor necrosis and survival [7, 17–22]. In the setting of HCC, disease progression following locoregional therapy is often associated with poor prognosis [26–28]. For example, studies have shown that HCC tumor progression measured by mRECIST following locoregional therapy is an independent risk factor of tumor recurrence and decreased survival following liver transplantation [26, 29]. Similarly, a study by De Carlis et al. demonstrated high recurrence rates and worse outcomes in patients with HCC progression while on the transplant wait list [27]. Recently the ART score was developed to aid in the decision making process for repeat TACE [28]. This scoring system predicts a poor prognosis following repeat TACE in patients with features of progressive HCC following initial TACE [28]. Predictive variables include a lack of radiologic response to initial TACE [28].

While this study does not address which HCC patients may benefit from an initial TACE, the data may inform treatment recommendations for patients after their first TACE procedure. Current HCC treatment goals focus on tumor eradication. However, the findings presented here suggest only a modest increase in median survival going from stable disease to partial and complete response. Perhaps the future paradigm should focus more on avoiding the progressive disease category, more of a “HCC treatment as a chronic disease” mindset instead of the (often unrealistic) goal of tumor eradication. Patients in the stable disease or partial response categories are the most common patients to undergo repeat TACE. These findings question the utility in giving these patients repeat TACE since only modest increases in median survival are seen with additional tumor necrosis. At the very least, it may be prudent not to rush to repeat TACE when a patient may require aggressive (nonselective) lobar embolization, in the setting of post-TACE liver dysfunction, or if a patient is on the liver transplant waitlist. In contrast, progressive disease as defined by mRECIST seems to carry an especially poor prognosis. The best approach for these patients may be alternative HCC treatments or even best supportive care.

This study has limitations to consider when interpreting the data. The retrospective design and practice patterns at UAB may bias the data. Well over half of the patients in this study (60%) were Child-Pugh B/C patients, which may not be representative of many patients receiving TACE. For example, studies included in a commonly referenced meta-analysis had patient populations with Child-Pugh A making up 70–100% [30]. Another practice pattern at UAB that may bias the data is that selective TACE procedures are commonly performed whereas nonselective lobar approaches are most common nationwide [31].

In conclusion, both the dichotomized (responders and nonresponders) and the four defined mRECIST responses to TACE in patients with HCC were predictive of survival. The main driver of this survival benefit analysis was the poor survival in the progressive disease group. Surprisingly, there was small nonsignificant survival benefit between complete response, partial response, and stable disease. Progressive disease, increasing total tumor diameter, and non-Child-Pugh class A were independent predictors of post-TACE mortality. These findings may inform HCC treatment decisions following first TACE procedures.

## Figures and Tables

**Figure 1:**
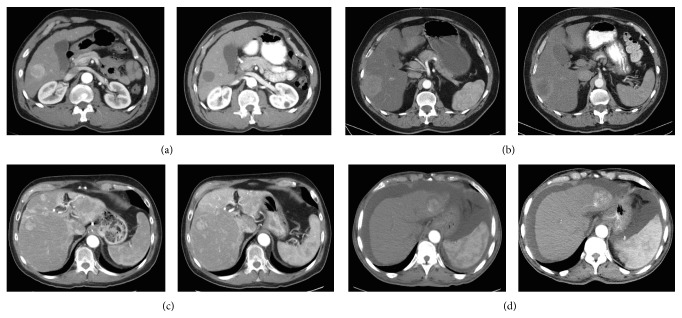
Axial CT images demonstrating the four mRECIST categories. (a) Complete: 100% HCC tumor necrosis. (b) Partial: 30%–99% HCC tumor necrosis. (c) Stable: between 29% HCC tumor necrosis and 20% HCC tumor growth. (d) Progressive: >20% HCC tumor growth.

**Table 1:** Baseline characteristics of patients with hepatocellular carcinoma treated with transarterial chemoembolization.

	mRECIST categories				*p* value
	Complete (*N* = 102)	Partial (*N* = 138)	Stable (*N* = 63)	Progressive (*N* = 14)	
Age (mean ± SD)	61.5 ± 8.7	61.5 ± 10.1	63.1 ± 9.5	61.9 ± 8.3	0.697

Gender					
Female	73 (71.6%)	110 (79.7%)	40 (71.4%)	10 (63.5%)	0.102
Male	29 (28.4%)	28 (20.3%)	23 (28.6%)	4 (36.5%)	

Race					
White	78 (76.5%)	105 (76.1%)	43 (68.3%)	7 (50.0%)	0.064
Black	18 (17.7%)	30 (21.7%)	13 (20.6%)	6 (42.9%)	
Other	6 (5.8%)	3 (2.2%)	7 (11.1%)	1 (7.1%)	

Etiology					
Alcohol	29 (28.4%)	35 (25.4%)	13 (21.0%)	2 (14.3%)	0.563
HBV	7 (6.9%)	6 (4.4%)	5 (7.9%)	2 (14.3%)	0.434
HCV	57 (55.9%)	73 (52.9%)	21 (33.9%)	5 (35.7%)	0.024
NASH	22 (21.6%)	28 (20.3%)	10 (15.9%)	1 (7.1%)	0.402

Child-Pugh class					0.525
A	35 (34.3%)	53 (38.4%)	28 (44.4%)	7 (50.0%)	
B	54 (52.9%)	74 (53.6%)	28 (44.4%)	7 (50.0%)	
C	13 (12.8%)	11 (8.0%)	7 (11.2%)	0 (0.0%)	

AFP^*∗*^	12.3 ± 100.8	22.0 ± 158.9	27.0 ± 132.6	132.0 ± 3601.0	0.179^*∗∗*^

Diameter of largest tumor	3.6 ± 1.8	4.6 ± 2.5	5.6 ± 4.4	6.1 ± 3.7	<0.0001

Number of tumors	1.6 ± 0.8	1.5 ± 1.0	2.1 ± 1.4	1.9 ± 1.6	0.003

^*∗*^Medians and interquartile range reported.

^*∗∗*^Kruskal-Wallis procedure used.

TACE: transarterial chemoembolization; mRECIST: modified Response Evaluation Criteria In Solid Tumors, *p* value: probability, SD: standard deviation, HBV: hepatitis B virus, HCV: hepatitis C virus, NASH: nonalcoholic steatohepatitis, and AFP: alpha fetoprotein.

**Table 2:** Multivariable analyses of post-TACE survival.

	Univariate			Multivariable		
	Hazard ratio	95% CI	*p* value	Hazard ratio	95% CI	*p* value
Age	0.99	(0.98, 1.01)	0.500	1.00	(0.98, 1.02)	0.721

Gender			0.295^*∗∗*^			0.167^*∗∗*^
Male	1.00 (reference)	(—, —)		1.00 (reference)	(—, —)	
Female	1.23	(0.84, 1.79)	0.295	1.35	(0.88, 2.06)	0.167

Race			0.108^*∗∗*^			0.132^*∗∗*^
White	1.00 (reference)	(—, —)		1.00 (reference)	(—, —)	
Black	0.88	(0.58, 1.36)		0.75	(0.47, 1.19)	0.222
Other	1.91	(0.99, 3.68)		1.57	(0.80, 3.08)	0.194

Child-Pugh			<0.0001^*∗∗*^			<0.0001^*∗∗*^
A	1.00 (reference)	(—, —)		1.00 (reference)	(—, —)	
B	2.17	(1.50, 3.15)	<0.001	2.67	(1.80, 3.94)	<0.001
C	2.09	(1.05, 4.16)	0.037	2.26	(1.09, 4.65)	0.028

Total tumor diameter^*∗*^	1.04	(1.01, 1.07)	0.009	1.04	(1.01, 1.08)	0.009

mRECIST			0.034^*∗∗*^			0.003^*∗∗*^
Complete	1.00 (reference)	(—, —)		1.00 (reference)	(—, —)	
Partial	1.15	(0.77, 1.72)	0.499	1.18	(0.85, 2.14)	0.436
Stable	1.39	(0.83, 2.33)	0.209	1.28	(0.55, 2.03)	0.379
Progressive	3.21	(1.42, 7.24)	0.005	4.99	(1.19, 11.09)	<0.0001

^*∗*^Sum axial diameter of three largest hepatocellular carcinoma tumors.

^*∗∗*^Multiple degree of freedom test to determine if any of the levels within the categorical variables differs from the reference group within that variable.

TACE: transarterial chemoembolization; mRECIST: modified Response Evaluation Criteria In Solid Tumors, *p* value: probability, and 95% CI: 95% confidence interval.
